# Perceived Emotional Support and Burnout Dimensions Among Physical Therapists: A Cross‐Sectional Study

**DOI:** 10.1002/pri.70345

**Published:** 2026-09-16

**Authors:** Michal Klein, Yael Krebs, Merav Ben Natan

**Affiliations:** ^1^ University of Haifa Haifa Israel; ^2^ Maccabi Health Services Tel Aviv Israel; ^3^ Department of Nursing Tel Aviv University Tel Aviv Israel; ^4^ Hillel Yaffe Academic School of Nursing Hillel Yaffe Medical Center Hadera Israel

**Keywords:** burnout, emotional exhaustion, emotional support, physical therapists, social support

## Abstract

**Background and Purpose:**

Burnout may differ across its dimensions, yet the role of perceived emotional/social support among physical therapists remains unclear. This study examined burnout dimensions, their associations with perceived support, and physical therapists' reported support needs in Israel.

**Methods:**

A cross‐sectional online survey was completed by 207 licensed physical therapists. Burnout was assessed using the Maslach Burnout Inventory – Human Services Survey for Medical Personnel, and support was assessed using an adapted work‐related scale. Associations were examined using bivariate analyses and multivariable linear regression adjusted for age group, sex, and work setting. Open‐ended responses were analyzed descriptively.

**Results:**

Elevated emotional exhaustion was identified in 28.9% of participants, elevated depersonalization in 14.4%, and low personal accomplishment in 19.3%. Higher support was associated with lower emotional exhaustion (*B* = −1.60, 95% CI [−2.76, −0.43], *p* = 0.008) and higher personal accomplishment (*B* = 2.14, 95% CI [1.45, 2.84], *p* < 0.001), but not with depersonalization. Participants primarily requested structured professional and peer support.

**Discussion:**

The findings demonstrate a dimension‐specific pattern: perceived support was associated with lower emotional exhaustion and higher personal accomplishment but not with depersonalization. Interpersonal support alone may therefore be insufficient to address all dimensions of burnout, and organizational working conditions may require separate attention.

## 1 Introduction

Burnout is a work‐related syndrome that develops in response to chronic emotional and interpersonal stressors and is commonly characterized by emotional exhaustion, depersonalization, and reduced personal accomplishment (Maslach et al. 2001). Originally conceptualized in people‐oriented occupations, burnout remains particularly relevant across helping professions, including health care, human services, and education, where work involves sustained emotional engagement and repeated exposure to others' needs and distress (Maslach and Leiter 2016). In health care, burnout is both a workforce and quality‐of‐care concern, as it may affect clinicians' engagement, retention, and capacity to provide consistent patient‐centered care (Hodkinson et al. 2022).

Physical therapy represents a particularly relevant helping profession because physical therapists' work requires repeated interpersonal contact, emotional responsiveness, and ongoing adaptation to patients' pain, functional limitations, uncertainty, and psychological distress (McGrath et al. 2024). A recent systematic review and meta‐analysis of 32 studies including 5984 physical therapists from 17 countries estimated a pooled burnout prevalence of 8%, whereas 27% reported emotional exhaustion, 23% reported depersonalization, and 25% reported low personal accomplishment (Venturini et al. 2024). This variation supports examining burnout through its distinct dimensions rather than solely as a global syndrome.

The Job Demands–Resources model provides a theoretical framework for understanding burnout across helping professions (Bakker and Demerouti 2017; Demerouti et al. 2001). According to this model, burnout may develop when demands such as workload, documentation requirements, limited autonomy, and emotional exposure outweigh the resources available to address them. Across healthcare occupations, recent prospective meta‐analytic evidence indicates that job demands—including workload, role stress, and patient‐related threats—are associated with poorer employee well‐being, whereas job resources such as control and support may protect against subsequent strain (Marzocchi et al. 2024). Evidence among nurses, physicians, and allied health professionals similarly indicates that social and organizational support may reduce burnout and promote well‐being (Cohen et al. 2023; Velando‐Soriano et al. 2020). These findings provide a broader theoretical basis for examining emotional/social support as a potential resource in physical therapy.

Although job resources have been associated with burnout among physical therapists, evidence regarding whether perceived emotional/social support relates similarly to each burnout dimension remains limited (Patel and Bartholomew 2021; Venturini et al. 2024). Because the burnout dimensions may reflect different underlying processes, emotional/social support may be more closely related to emotional exhaustion and personal accomplishment, whereas depersonalization may be more strongly influenced by workplace and organizational conditions (Cantu et al. 2022; Venturini et al. 2024). Recent evidence suggests that organizational and peer support may strengthen physical therapists' satisfaction derived from helping patients and thereby reduce their intention to leave (Roitenberg and Ben‐Ami 2025). Evidence from Israel remains limited. Therefore, this study aimed to describe the three burnout dimensions, examine their associations with perceived emotional/social support, and explore the types of support physical therapists report needing. Based on the Job Demands–Resources model, we hypothesized that higher perceived emotional/social support would be associated with lower emotional exhaustion and depersonalization and higher personal accomplishment.

## 2 Methods

### 2.1 Participants

This cross‐sectional online survey was conducted among licensed physical therapists working in Israel. Eligible participants were physical therapists aged 20 years or older who were currently working in the profession and were able to complete the online questionnaire in Hebrew.

A total of 207 eligible physical therapists completed the questionnaire and constituted the overall study sample. Sample size was estimated a priori for detecting the primary hypothesized associations between perceived emotional/social support and the burnout dimensions. For a two‐tailed Pearson correlation, assuming a small‐to‐moderate effect size of *r* = 0.20, *α* = 0.05, and 80% power, the required sample size was 194 participants. A correlation‐based calculation was selected because the available literature did not provide sufficiently reliable estimates of the incremental variance attributable to perceived support after adjustment for the planned covariates.

### 2.2 Procedures

Data were collected between March 2026 and May 2026 using an anonymous self‐administered online questionnaire. The survey was distributed digitally to physical therapists in Israel through professional networks. Because the invitation could be forwarded through these networks, the total number of individuals who received or viewed it could not be determined; therefore, a response rate was not calculated. The first page of the survey presented information about the study purpose, voluntary participation, anonymity, confidentiality, and the right to withdraw at any time without consequences. Participants were informed that completion of the questionnaire indicated their consent to participate. No identifying information was collected. The questionnaire required approximately 10–15 min to complete. Data were exported from the online survey platform into a password‐protected electronic database for statistical analysis. The study was approved by the Institutional Review Board of Hillel Yaffe Medical Center, Hadera, Israel (approval no. 0064‐26‐HYMC) and was conducted in accordance with the Declaration of Helsinki. Reporting was guided by the STROBE statement.

### 2.3 Measures

#### 2.3.1 Demographic and Professional Characteristics

Participants reported demographic and professional characteristics, including age group, sex, marital status, number of children, geographic region, professional education, main field of physical therapy practice, years of professional experience, primary work setting, and employment scope. For analysis, work settings were categorized as hospitals or medical centers, health maintenance organizations, private practice or home care, rehabilitation or long‐term care settings, and education or child‐development settings.

#### 2.3.2 Perceived Emotional/Social Support

Perceived emotional/social support related to work was assessed using a contextually adapted Hebrew version of the 12‐item Multidimensional Scale of Perceived Social Support (MSPSS) (Zimet et al. 1990). The MSPSS was selected because it assesses perceived support from three interpersonal sources—significant others, family, and friends—which may provide important emotional resources when coping with work‐related challenges. A forward‐ and back‐translation procedure was used to support linguistic and conceptual equivalence. The translated and contextually modified items were reviewed by a panel of three experts in physical therapy and burnout for clarity, relevance, and conceptual equivalence. These procedures supported the linguistic and contextual appropriateness of the adaptation but were not intended to establish its construct validity or broader psychometric properties. Accordingly, the instrument is described as a contextually adapted work‐related measure rather than as a validated work‐related version of the MSPSS.

The original 12‐item structure and three support‐source domains—significant‐other, family, and friend support—were retained, with four items representing each domain. The wording was contextually modified to explicitly refer to work‐related or professional challenges. For example, the original item “I can count on my friends when things go wrong” was adapted to “I can count on my friends when problems arise in my work as a physical therapist.” Responses were rated from 1 (very strongly disagree) to 7 (very strongly agree), with higher scores indicating greater perceived support. Mean scores were calculated for the total scale and each source‐specific subscale. In the present sample, internal consistency was excellent for the total adapted scale (Cronbach's *α* = 0.93), significant‐other support (*α* = 0.89), family support (*α* = 0.92), and friend support (*α* = 0.91). These internal‐consistency estimates reflect score reliability in the present sample and should not be interpreted as evidence of construct validity.

#### 2.3.3 Burnout

Burnout was assessed using the Maslach Burnout Inventory–Human Services Survey for Medical Personnel (MBI‐HSS [MP]; Maslach et al. 1996). This 22‐item version was selected because it was developed for medical and helping professionals who provide direct patient care. It assesses emotional exhaustion, depersonalization in relation to care recipients, and personal accomplishment, which are particularly relevant to physical therapists because their work involves sustained interpersonal and emotionally demanding contact with patients. Its use also facilitates comparison with previous physical therapy research, in which the MBI has been the most frequently used burnout instrument (Venturini et al. 2024). The response categories are 0 (never), 1 (a few times a year or less), 2 (once a month or less), 3 (a few times a month), 4 (once a week), 5 (a few times a week), and 6 (every day). The emotional exhaustion subscale includes 9 items and reflects feelings of being emotionally depleted by work. The depersonalization subscale includes 5 items and reflects detached or impersonal responses toward recipients of care. The personal accomplishment subscale includes 8 items and reflects perceived competence and achievement at work.

Items were summed to create each subscale score. Higher emotional exhaustion and depersonalization scores indicate higher burnout, whereas lower personal accomplishment scores indicate higher burnout. In the present sample, internal consistency was excellent for emotional exhaustion (Cronbach *α* = 0.910), acceptable for depersonalization (Cronbach *α* = 0.727), and good for personal accomplishment (Cronbach *α* = 0.828). Commonly used MBI cutoffs were applied descriptively to characterize burnout levels: emotional exhaustion scores of 27 or higher, depersonalization scores of 10 or higher, and personal accomplishment scores of 33 or lower.

#### 2.3.4 Open‐Ended Support Question

Participants were also invited to answer the following optional open‐ended question: “What type of support would you like to receive in order to cope better with the challenges that arise around your work and profession?” Responses were inductively coded into recurring categories and summarized as frequencies and percentages. This exploratory analysis complemented the quantitative findings and was not intended as a full qualitative analysis.

### 2.4 Data Analysis

Categorical variables were summarized as frequencies and percentages and continuous scores as means and standard deviations. Internal consistency was assessed using Cronbach's alpha. Available‐case analysis was used, with scores calculated for participants completing the relevant scale or subscale. Associations were examined using Pearson or Spearman correlations, independent‐sample *t* tests, or analysis of variance, as appropriate.

Separate linear regression models examined associations between perceived support and each burnout dimension, adjusting for age group, sex, and dummy‐coded work setting, with private practice/home care as the reference. Covariates were selected a priori as potential confounders, with age and work setting also supported by observed associations with burnout. In the primary models, age group was entered as an ordinal predictor coded 1 = 20–30, 2 = 31–40, 3 = 41–50, 4 = 51–60, and 5 = ≥ 61 years. To assess the robustness of this specification, additional sensitivity models categorically treated the age group, with the 31–40‐year group as the reference category. Because age and professional experience both represent career stage, sensitivity models also substituted professional experience for age to limit multicollinearity. Assumptions were assessed using residual plots and variance inflation factors. Unstandardized coefficients, 95% confidence intervals, *p* values, and *R* ^2^ are reported. Analyses used IBM SPSS Statistics, version 29, with *p* < 0.05.

## 3 Results

### 3.1 Sample Characteristics

A total of 207 physical therapists completed the survey. The sample was predominantly female and included mainly experienced clinicians, with health maintenance organizations and private practice/home care as the most common work settings (Table 1).

**TABLE 1:** Sample description (*N* = 207).

Characteristic	*n*	%
Sex		
Female	161	77.8
Male	46	22.2
Age group		
20–30	4	1.9
31–40	71	34.3
41–50	78	37.7
51–60	39	18.8
≥ 61	15	7.3
Marital status		
Single	17	8.2
Married/in a relationship	174	84.1
Divorced	15	7.2
Widowed	1	0.5
Professional education		
Bachelor’s degree	124	59.9
Master’s degree	79	38.2
Doctoral degree or higher	4	1.9
Years of professional experience		
< 1 year	3	1.4
1–5 years	8	3.9
6–10 years	34	16.4
11–15 years	58	28.0
16–20 years	38	18.4
> 20 years	66	31.9
Main field of physical therapy		
Orthopedics	86	41.5
Pediatrics/child development	58	28.0
Neurology/rehabilitation	33	15.9
Geriatrics	14	6.8
Respiratory	5	2.4
Pelvic floor	5	2.4
Other	6	2.9
Work setting		
Health maintenance organization	61	29.5
Private practice/home care	59	28.5
Hospital/medical center	36	17.4
Rehabilitation/long‐term care	23	11.1
Education/child‐development settings	20	9.7
Other	8	3.9

*Note:* Percentages are based on the total sample unless otherwise indicated. Some categories include free‐text responses that were recoded into the closest substantive category.

### 3.2 Perceived Support and Burnout

Mean perceived emotional/social support was 5.12 (SD = 1.25). Mean scores were 21.10 (SD = 11.16) for emotional exhaustion, 4.81 (SD = 4.68) for depersonalization, and 38.70 (SD = 6.78) for personal accomplishment. Elevated emotional exhaustion, elevated depersonalization, and low personal accomplishment were identified in 28.9%, 14.4%, and 19.3% of participants, respectively. Among participants with complete MBI data, 34.6% had elevated emotional exhaustion and/or depersonalization, whereas 4.2% had elevations across all three dimensions.

### 3.3 Bivariate Associations

Older age and greater professional experience were associated with lower emotional exhaustion and depersonalization and higher personal accomplishment (all *p* ≤ 0.003). Sex was unrelated to burnout or perceived support. Emotional exhaustion and depersonalization differed by work setting, whereas personal accomplishment and perceived support did not.

Perceived support was associated with lower emotional exhaustion and higher personal accomplishment but not with depersonalization. Support from a significant other was associated with both emotional exhaustion and personal accomplishment, whereas family and friend support were associated only with personal accomplishment. Complete correlation coefficients are presented in Table 2.

**TABLE 2:** Pearson correlations between support sources and burnout dimensions.

Variable	Emotional exhaustion	Depersonalization	Personal accomplishment
Total perceived support	−0.14*	−0.09	0.37***
Support from a significant other	−0.17*	−0.10	0.35***
Family support	−0.13	−0.13	0.34***
Friend support	−0.05	0.01	0.25***
Emotional exhaustion	—	0.51***	−0.47***
Depersonalization		—	−0.32***

*Note:* Values are Pearson *r* coefficients.

*p < 0.05; **p < 0.01; ***p < 0.001. Higher personal accomplishment indicates lower burnout.

### 3.4 Adjusted Analyses

The overall regression models for emotional exhaustion (*N* = 197), depersonalization (*N* = 198), and personal accomplishment (*N* = 193) were each statistically significant, explaining 15.6%–22.8% of the variance in the respective burnout dimensions (Table 3). At the individual‐predictor level, perceived support was significantly associated with lower emotional exhaustion (*B* = −1.60, 95% CI [−2.76, −0.43], *p* = 0.008) and higher personal accomplishment (*B* = 2.14, 95% CI [1.45, 2.84], *p* < 0.001), whereas its coefficient for depersonalization was not statistically significant (*B* = −0.33, 95% CI [−0.83, 0.16], *p* = 0.187). Older age remained associated with all three dimensions, while work setting was associated with emotional exhaustion and depersonalization. Sex was not a significant predictor. Sensitivity analyses replacing the age group with professional experience or categorically treating the age group with the 31–40‐year group as the reference category did not materially alter the direction, magnitude, or statistical significance of the associations between perceived support and the burnout dimensions. Inspection of the residual plots indicated no substantial departures from linearity, homoscedasticity, or approximate normality. Variance inflation factors ranged from 1.03 to 1.53 across the models, indicating no problematic multicollinearity.

**TABLE 3:** Multiple linear regression models predicting burnout dimensions.

Outcome and predictor	*B*	95% CI	*p* value
Emotional exhaustion (*N* = 197)			
Perceived emotional/social support	−1.60	−2.76 to −0.43	0.008
Age group	−2.56	−4.21 to −0.92	0.002
Female sex	2.46	−1.31 to 6.23	0.200
Health maintenance organization	5.65	1.70 to 9.59	0.005
Hospital/medical center	5.86	1.28 to 10.44	0.012
Rehabilitation/long‐term care	3.50	−1.76 to 8.76	0.191
Education/child‐development settings	−1.44	−6.78 to 3.89	0.594
Depersonalization (*N* = 198)			
Perceived emotional/social support	−0.33	−0.83 to 0.16	0.187
Age group	−0.74	−1.43 to −0.05	0.036
Female sex	−1.32	−2.92 to 0.29	0.107
Health maintenance organization	1.88	0.21 to 3.55	0.027
Hospital/medical center	2.76	0.80 to 4.72	0.006
Rehabilitation/long‐term care	3.02	0.80 to 5.24	0.008
Education/child‐development settings	−1.15	−3.38 to 1.08	0.310
Personal accomplishment (*N* = 193)			
Perceived emotional/social support	2.14	1.45 to 2.84	< 0.001
Age group	1.56	0.56 to 2.56	0.002
Female sex	−1.13	−3.41 to 1.15	0.330
Health maintenance organization	−2.24	−4.59 to 0.11	0.062
Hospital/medical center	−2.36	−5.15 to 0.43	0.097
Rehabilitation/long‐term care	−2.79	−5.90 to 0.33	0.079
Education/child‐development settings	−2.56	−5.69 to 0.57	0.108

*Note:* Separate linear regression models were conducted for each burnout dimension. Analytic sample sizes varied because available‐case analysis was conducted separately for each burnout outcome. Analytic sample sizes varied because available‐case analysis was used for each outcome. Age group was entered as an ordinal variable coded 1 = 20–30, 2 = 31–40, 3 = 41–50, 4 = 51–60, and 5 = ≥ 61 years. Sex was coded as female versus male. Work setting was entered using dummy variables, with private practice/home care as the reference category. The model for emotional exhaustion was significant, *R* ^2^ = 0.170, *F*(7, 189) = 5.52, *p* < 0.001. The model for depersonalization was significant, *R* ^2^ = 0.156, *F*(7, 190) = 5.01, *p* < 0.001. The model for personal accomplishment was significant, *R* ^2^ = 0.228, *F*(7, 185) = 7.80, *p* < 0.001. Higher personal accomplishment indicates lower burnout.

Abbreviations: *B* = unstandardized regression coefficient; CI = confidence interval.

### 3.5 Open‐Ended Support Needs

Of the full sample, 112 participants (54.1%) responded to the optional open‐ended question. Among these respondents, 53 (47.3%) requested structured professional emotional support, and 45 (40.2%) requested peer‐ or team‐based support. Managerial or organizational support was identified by 20 respondents (17.9%), and workload‐related changes by 14 (12.5%). Eight respondents (7.1%) reported no additional support needs. Categories were not mutually exclusive.

## 4 Discussion

This study aimed to examine burnout dimensions among physical therapists in Israel, their associations with perceived emotional/social support, and physical therapists' reported support needs. The most clinically relevant finding was the dimension‐specific pattern: higher perceived support was associated with lower emotional exhaustion and higher personal accomplishment but not with depersonalization. This finding reinforces the view that burnout is multidimensional and that its components may have distinct determinants and require different preventive approaches (Maslach et al. 2001; Venturini et al. 2024).

Emotional exhaustion appeared to be the most salient expression of burnout‐related strain. This is plausible because physical therapy is a helping profession requiring sustained interpersonal engagement and repeated exposure to patients' pain, distress, and uncertain recovery (McGrath et al. 2024). Within the Job Demands–Resources framework, such demands are more likely to produce exhaustion when adequate interpersonal and organizational resources are lacking (Bakker and Demerouti 2017; Demerouti et al. 2001). Assessing burnout by dimension may therefore help identify meaningful emotional strain that would be missed by relying solely on a global burnout classification.

In standardized terms, the association was small for emotional exhaustion and small‐to‐moderate for personal accomplishment. This suggests that support may contribute more to preserving competence, professional meaning, and engagement than to eliminating the emotional demands of clinical work. Supportive relationships may provide validation and opportunities to process difficult experiences, consistent with evidence that social resources buffer occupational strain and help preserve satisfaction derived from helping patients (Diehl et al. 2021; Roitenberg and Ben‐Ami 2025; Velando‐Soriano et al. 2020).

The absence of an adjusted association with depersonalization is also informative. Personal support may facilitate emotional recovery but cannot necessarily alter workplace conditions that encourage psychological distancing. The differences across work settings similarly suggest that organizational context‐including workload, staffing, documentation demands, productivity expectations, and professional autonomy‐may be particularly relevant to this burnout dimension. Emotional/social support should therefore complement, rather than replace, organizational interventions (Cantu et al. 2022; Carmona‐Barrientos et al. 2020; Patel and Bartholomew 2021).

Different support sources may also serve different functions. A significant other may provide a safe context for processing difficult professional experiences, whereas family and friends may contribute more broadly to encouragement and professional confidence. However, these distinctions remain preliminary because the support measure was adapted to the work context and requires further validation (Zimet et al. 1990).

The associations with age and professional experience may reflect increasing confidence, improved coping, and clearer emotional boundaries across a professional career. Alternatively, physical therapists experiencing substantial burnout may move to less demanding positions or leave the profession, producing a possible healthy‐worker effect. These explanations cannot be distinguished in a cross‐sectional study but suggest that early‐career physical therapists may particularly benefit from mentoring, clinical supervision, and structured professional reflection (Çelik and Şevgin 2025; Elinich et al. 2024).

The open‐ended responses suggest potential elements of a multilevel approach to workforce support. Peer‐reflection groups, mentoring, clinical supervision, professional debriefing, and accessible psychological consultation should be combined with managerial recognition, flexibility, and attention to workload, staffing, and documentation demands. Such an approach is consistent with evidence that interventions addressing both staff support and working conditions may be more useful than those focused solely on individual coping (Cohen et al. 2023; Razai et al. 2023; Snowdon et al. 2020). However, these suggestions should be considered exploratory, as their effectiveness was not evaluated in the present study. Prospective studies are needed to evaluate whether these strategies reduce subsequent burnout.

Although conducted in Israel, the processes examined may also be relevant across healthcare systems. International evidence indicates that physical therapists experience similar relational and organizational demands, although their intensity and consequences may vary according to local workforce structures and resources (Cantu et al. 2022; Carmona‐Barrientos et al. 2020; Venturini et al. 2024). Physical therapy services in different countries may therefore benefit from combining interpersonal support with organizational interventions tailored to their local working conditions.

## 5 Limitations

The cross‐sectional design precludes causal inference and cannot rule out reverse causation between perceived support and burnout. Convenience and snowball sampling may limit representativeness, and self‐reporting measures are susceptible to response and social desirability biases. Small work‐setting subgroups may have reduced the precision of comparisons. The achieved sample exceeded the a priori target; however, the sample‐size calculation was based on a bivariate correlation rather than on the final multivariable regression models. Consequently, the study was not specifically powered to detect the incremental contribution of perceived support within each adjusted model. Although the contextually adapted work‐related support measure demonstrated high internal consistency in the present sample, it was not subjected to formal psychometric validation. Expert review and internal‐consistency estimates did not establish construct validity, and the measure's factor structure, convergent and discriminant validity, and test–retest reliability were not examined. Findings involving perceived support should therefore be interpreted cautiously as being based on a contextually adapted, but not fully validated, work‐related measure. In addition, the open‐ended findings remain exploratory. Residual confounding by unmeasured occupational factors, including workload, staffing, documentation demands, and organizational culture, is also possible.

## 6 Implications for Physical Therapy Practice

Burnout assessment should consider its separate dimensions rather than rely solely on a full burnout profile, as meaningful emotional exhaustion may occur without concurrent elevation in the other dimensions. Workforce strategies should combine peer discussion, mentoring, clinical supervision, and professional reflection with organizational attention to workload, staffing, flexibility, documentation demands, managerial recognition, and team culture. Interpersonal support alone should therefore not be viewed as sufficient to address all dimensions of burnout. Emotional/social support may help preserve emotional resources and professional accomplishment, whereas reducing depersonalization may require more direct changes to workplace conditions.

## Author Contributions

**Michal Klein:** conceptualization, investigation, data curation, writing – review and editing. **Yael Krebs:** conceptualization, investigation, data curation, writing – review and editing. **Merav Ben Natan:** methodology, supervision, formal analysis, writing – original draft, writing – review and editing. All authors approved the final manuscript and agree to be accountable for all aspects of the work.

## Funding

The authors have nothing to report.

## Ethics Statement

The study was approved by the Institutional Review Board of Hillel Yaffe Medical Center, Hadera, Israel (approval no. 0064‐26‐HYMC; approval date: April 29, 2026).

## Consent

The study did not involve patients. All participants were licensed physical therapists. Participation was voluntary and anonymous, and completion of the questionnaire after reviewing the study information was considered informed consent to participate.

## Conflicts of Interest

The authors declare no conflicts of interest.

## Permission to Reproduce Material From Other Sources

The authors have nothing to report.

## Data Availability

The data that support the findings of this study are available from the corresponding author upon reasonable request and subject to ethical and institutional restrictions.
